# Interactive influences of fluctuations of main food resources and climate change on long-term population decline of Tengmalm’s owls in the boreal forest

**DOI:** 10.1038/s41598-020-77531-y

**Published:** 2020-11-24

**Authors:** Marek Kouba, Luděk Bartoš, Jitka Bartošová, Kari Hongisto, Erkki Korpimäki

**Affiliations:** 1 Section of Ecology, Department of Biology, University of Turku, Turku, Finland; 2 Department of Ethology and Companion Animal Science, Faculty of Agrobiology, Food and Natural Resources, Czech University of Life Sciences Prague, Prague, Czech Republic; 3 Department of Ethology, Institute of Animal Science, Prague, Czech Republic; 4 Unaffiliated, Tampere, Finland

**Keywords:** Zoology, Ecology, Boreal ecology

## Abstract

Recent wildlife population declines are usually attributed to multiple sources such as global climate change and habitat loss and degradation inducing decreased food supply. However, interactive effects of fluctuations in abundance of main foods and weather conditions on population densities and reproductive success have been studied rarely. We analysed long-term (1973–2018) data on Tengmalm’s owl (*Aegolius funereus*) and the influence of prey abundance and weather on breeding densities and reproductive success in western Finland. We found that fledgling production per breeding attempt declined and laying date of the owl population delayed during the period between 1973 and 2018. The breeding density of the owl population decreased with increasing temperature in winter (October–March), fledgling production increased with increasing temperature and precipitation in spring (April–June), whereas the initiation of egg-laying was delayed with increasing depth of snow cover in late winter (January–March). The decreasing trend of fledgling production, which was mainly due to starvation of offspring, was an important factor contributing to the long-term decline of the Tengmalm’s owl study population. Milder and more humid spring and early summer temperatures due to global warming were not able to compensate for lowered offspring production of owls. The main reason for low productivity is probably loss and degradation of mature and old-growth forests due to clear-felling which results in loss of coverage of prime habitat for main (bank voles) and alternative foods (small birds) of owls inducing lack of food, and refuges against predators of Tengmalm’s owls. This interpretation was also supported by the delayed start of egg-laying during the study period although ambient temperatures increased prior to and during the egg-laying period.

## Introduction

Wildlife population declines are nowadays reported all over the world and encompass most major taxonomic groups across most possible habitats and environments on the planet^1^. However, causes governing these declines are seldom unambiguous and are mostly attributed to multiple sources such as global climate change^2–4^, as well as habitat loss and degradation due to land-use changes, e.g., intensive agricultural practices, deforestation and clear-cutting of forests^5–12^. Additional species declines due to the above-mentioned causes and their interactions can be expected^13^, but future changes and their effects on wildlife populations are not simple to predict because factors affecting population sizes are complex and include intrinsic and extrinsic factors^14,15^. In addition, taxa vary in their vulnerability to climate change and habitat changes^7,16^.

Weather can modify densities of animal populations via changes in reproductive success and survival, but also by modifying habitat and food availability^17^. In recent years, large-scale climatic changes have been causing declines in populations of many species, by reducing reproductive success due to, for example, phenological mismatches thus declining population abundance to even local extinctions^18^. Temperatures have been rising world-wide, but increases in temperature have been larger at northern latitudes and during winter and spring^19,20^. Furthermore, precipitation at northern latitudes has also increased due to both temperature changes and altered air circulation patterns.

Food abundance and weather conditions are key factors influencing the reproductive success, survival and population densities of birds and other animals^17,21^. The relative importance of food and weather for reproduction of birds has rarely been investigated in the same study (but see^22–25^). Birds of prey are commonly studied for food limitation on population densities, breeding performance and reproductive success because the abundance and availability of their main foods can be relatively accurately estimated in the field^26^. Firm evidence for food limitation on population densities and reproductive success of birds of prey subsisting on fluctuating food resources (e.g., small rodents and lagomorphs) have been demonstrated by several research groups in boreal and arctic regions^26–32^. Climate-induced changes in species distribution and population densities are attributable to changes in the demography of local population, i.e. the sum of the influence on breeding success, survival and dispersal^24^. Breeding success can be expected to be particularly important because young individuals are usually more prone to disperse than adults^33^. Since weather may affect breeding success directly, for example through alteration in the survival of offspring caused by changing weather conditions^34,35^, or indirectly through the food chain due to changes in food or predator abundances^36,37^, it is essential to determine the relative importance of food supply and direct weather conditions on reproductive success.

In this study, we focused on a population of Tengmalm’s owls (*Aegolius funereus*), a small nocturnal cavity-nesting owl (body mass of males approx. 100 g and that of females approx. 150 g) living in coniferous forests in the boreal zone and in alpine forests further south in the Holarctic region^38,39^. They feed mainly on small mammals, among which voles of the genera *Myodes* and *Microtus* are their main prey and shrews of the genus *Sorex* and small forest birds are their most important alternative prey^40–43^. A male owl provides most of the food to a female and their owlets from prior to egg-laying until the fledglings become independent at the age of five to nine weeks^40,44–46^. The female incubates the eggs, broods the young, and remains almost continually in the nest cavity until the young are about 3 weeks old^40,47^.

Abundance of main foods (voles) is the key factor that governs the life of Tengmalm’s owls including the start of egg-laying, clutch size, breeding success, survival and dispersal^42^. The habitat composition within a home range also plays an important role^48–50^. Over-winter survival and lifetime reproductive success of male owls increased in home ranges that included high proportions of old-growth forest, although the extent of old-growth forest within their territories was relatively small, covering only 12% on average^48,50^. However, the impacts of fluctuating abundance of main foods and climate change on population dynamics, timing of breeding and reproductive success of owls and other birds of prey have been rarely analysed. For instance, in two relatively large owl species (the Ural owl *Strix uralensis* and the tawny owl *S. aluco*) breeding performance and climatic conditions were investigated in Finland, thus in the same environmental conditions as our study took place. In both species increasing late winter or early spring temperatures accelerated breeding at least as much as did high autumn abundance of main foods (voles); these temperature changes did not modify hatching dates and brood sizes of two smaller owl species, Tengmalm’s owl and the Eurasian pygmy owl *Glaucidium passerinum*^24^. Increasing snow depth in March delayed initiation of egg-laying in Ural owls and augmented the brood size of Tengmalm’s owls, but brood size was mainly determined by spring vole abundance in all four owl species^24^. The probability of breeding and number of fledglings of tawny owls were higher in years with high abundance of voles and in cold and dry winters^25^.

The nation-wide population of Tengmalm’s owls in Finland has shown a 2% annual decline from the 1980s to 2010s resulting in an overall decrease of the population by about 70% from 1980 up to 2019^42^. These population declines may be due to either reduced reproductive success and/or decreased adult and juvenile survival. We first analysed whether the long-term trend of Tengmalm’s owl population within our study area is attributable to changes in main foods and weather conditions. We investigated reproductive performance, i.e. breeding density estimate (number of nests/100 nest boxes), clutch size, yearly numbers of fledglings produced per breeding attempt, and laying date in relation to densities of main foods and weather variables during a 46-year period from 1973–2018. This unique data set offered us the opportunity to investigate whether (1) breeding density estimate, (2) clutch size and (3) the number of fledglings produced per breeding attempt have decreasing trends over the entire study period and whether interactive effects of abundance of main foods and weather conditions are partly inducing population declines. On the basis of earlier knowledge^42^, we expected that the timing of egg-laying could show one of two opposing trends. Firstly, there could be no marked long-term trend in the timing of egg-laying. Secondly, the start of egg-laying could be earlier if connected with higher temperatures and less snow cover due to global warming in boreal regions (as found, for instance, in the common buzzard *Buteo buteo* and the goshawk *Accipiter gentilis*^23,51^). On the other hand, the timing of egg-laying could also be delayed during 1970s–2010s, if the abundance of main foods has declined during the study period because the initiation of the egg-laying is delayed by one month in poor vole years^26,28^. We analysed (4) whether there are long-term trends in the initiation of egg-laying during the 46-year study period and whether they are related to variation in main food abundance and/or weather conditions. Because parental age and body condition of parent owls may influence the timing of breeding, clutch size and reproductive success of Tengmalm’s owls^52–54^, age of the female and male parents and their body condition indices were included in models of clutch size and laying date.

## Materials and methods

### Study area

We conducted the study in the Kauhava region of west-central Finland (approx. 63° N, 23° E). This lowland study area is only 30–120 m above sea level and around 61% of the study area is forested: in 1990 approximately 39% was categorised as young forest, 11% middle-aged (30–80 years) and 11% old-growth forests (˃ 80 years)^49^. Nearly all the forest areas are managed: first harvested by thinning when trees are 30–40 years old, and thereafter clear-cutting at intervals of 60–80 years. Clear-cut areas are seeded or saplings of Norway spruce *Picea abies* or Scots pine *Pinus sylvestris* are planted. Consequently, old-growth forests comprise in 2018 around 1% of the area. Clear-cut and sapling areas accounted for 6% of the study area (in 1990), and agricultural land (mainly crop fields and pasture) covered 25%, peatland bogs 2%, other (settlements, roads etc.) 3%, and water (lakes, rivers, streams) 2% of the area^42,55^.

**Figure 1:**
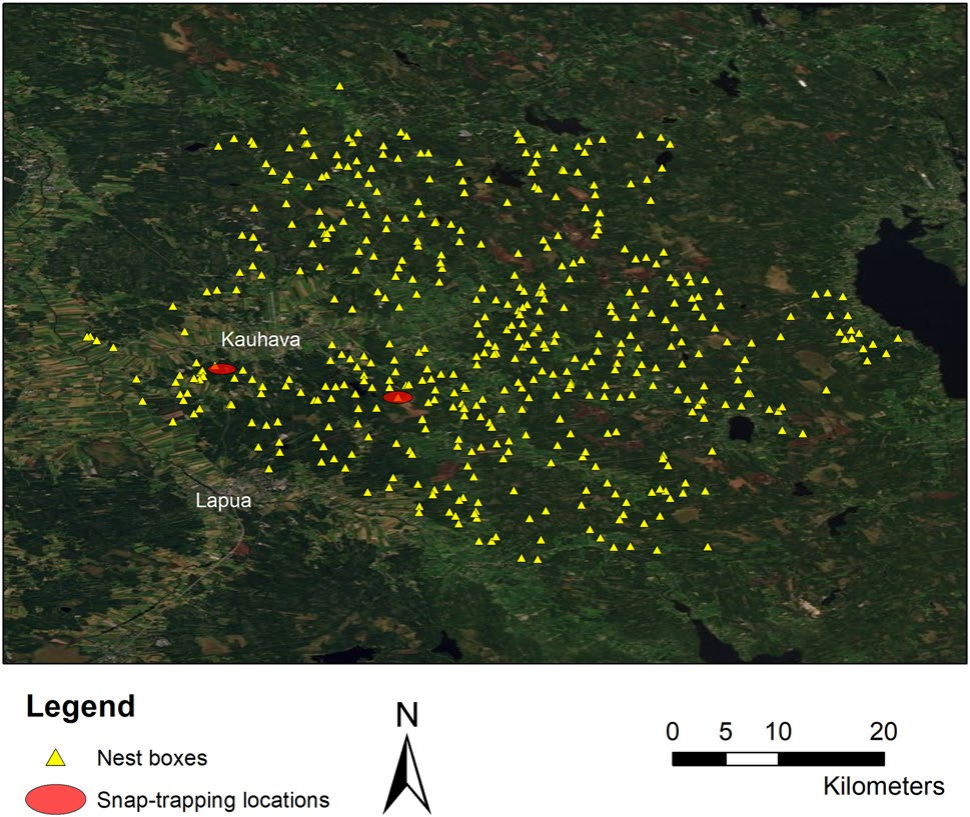
Map of the study area. Locations of nest boxes and natural cavities and two snap-trapping sites within the study area around the city of Kauhava, western Finland (ArcGIS version 10.5, https://www.esri.com; the map was created on the basis of a license granted to the Czech University of Life Sciences Prague). Background orthophoto map roughly shows borders of forest (dark green) and clear-cut, sapling and agricultural areas (light green and brown).

Tengmalm’s owls breed in the natural cavities made by black woodpeckers *Dryocopus martius* in Europe but readily accept nest boxes. The long-term study area covered 200 square km in 1973 and included 99 nest boxes. The increase of nest boxes and extension of the study area continued up to 1983, when the number of nest boxes reached 450 in an area of 1100 square km^42^. From 1983, the number of natural cavities found and checked annually has ranged between 20 and 30. We inspected these nest boxes and natural cavities (Fig. 1) annually during 1973–2018^42,55^.

We obtained meteorological data from the weather station located in the middle of the study area from the Finnish Meteorological Institute and included mean daily temperature (°C), total snow cover (cm), and daily precipitation (mm) during 1973–2018. Yearly means of weather data based on daily records (1973–2018) revealed increasing temperatures (Spearman rank correlation: r_s_ = 0.60, p < 0.001, n = 46) and precipitation (r_s_ = 0.32, p < 0.05, n = 46) as well as non-significant decreasing depth of snow cover (r_s_ = − 0.10, p = 0.53, n = 46). The average yearly temperatures increased by about two Celsius degrees during the study period. Long-term trends for weather variables and their individual periods used in particular analyses are presented in the Supporting Information file S1.

### Field procedures

We visited wooden nest boxes (18–20 × 18–20 cm base, 50–60 cm height and 8–10 cm diameter entrance hole) and natural cavities in the study area twice a year (late March to late April and mid-May to early June) to find nests. The date of the laying of the first egg was obtained mainly by back-dating from hatching dates using 29 days as the incubation period for the first-laid egg^40^. We inspected the nests sufficiently often to know the final number of eggs and hatchlings and to determine the hatching date (± 1 day). The age of the nestlings and their hatching order was in most cases based on the recorded date of hatching. In seldom cases where the exact date of hatching was not recorded, the ages of nestlings and their hatching order were estimated according to the growth curve of wing length valid for the studied population^40,42^. All nestlings were ringed, and from 1985 onwards weighed and the wing length measured at approximately the age of 25 days of the oldest owlet of the brood. We confirmed the number of fledglings by inspections after the breeding season when the presence or absence of the corpses and rings of succumbed nestlings were recorded. We trapped a vast majority of parent females and males breeding in the study area during the middle of the nestling period, ringed (if not re-trapped), aged by checking the moult of primary and secondary feathers^42^, and measured their wing length and body mass (females from 1977 and males from 1979 onwards; for trapping methods and measurement details, see^42^). Tengmalm’s owls can be reliably aged into three categories: 1-year, 2-year and older (3 +) owls^42^. In total, we recorded pieces of information about 1 761 nests and 3 971 nestlings during the study period and analysed in this study (for data see Supporting Information file S1).

We calculated the “scaled mass index” following Peig and Green^56^ to quantify the body mass relative to the body size of owls that allows to adjust the mass of all individuals to that which they would have if they had the same body size, using the equation of the linear regression of log-body mass on log-wing length estimated by regression. The regression slopes were 0.84 for males and 0.77 for females, whereas the average wing lengths were 171.7 and 178.9 mm, respectively. Thus, we calculated the scaled mass index (hereafter “BCI”, the body condition index), for instance, for adult males as body mass × (171.7/wing length)^0.84^ according to Peig and Green^56^. The wing length was routinely measured during our captures so that it served also as the proxy for skeletal body size.

We estimated abundances of main prey of Tengmalm’s owls (bank voles *Myodes glareolus*, field voles *Microtus agrestis* and sibling voles *M. rossiaemeridioinalis*) by snap-trapping each year in early May (i.e. breeding season) and in mid-September in both western and central parts of the study area (Fig. 1). Sampling was carried out in the four main habitat types (i.e. cultivated field, abandoned field, spruce forest, pine forest). Fifty-to-sixty baited Finnish metal mouse snap traps were set at 10 m intervals in vole runways on each sample plot and were checked daily for 3 consecutive days. The area of a sample plot was 0.5–0.6 ha and the pooled trapping effort was approx. 600 trap-nights in both western and central parts of the study area each year and season starting in 1973. The number of voles captured was standardized to the number of animals caught per 100 trap-nights to obtain the vole abundance index in the analyses (see^57^ for more details on trapping methods and vole cycles in the study area). As found earlier^57,58^, densities of bank and *Microtus* voles fluctuate in synchrony in the study area and the regional synchrony of vole population cycles extends up to 80 km, i.e. to the whole study area (see the Supporting Information file S1 for detailed abundance of main prey in different study years).

All experimental protocols were approved by the Finish Museum of National History (ringing licence no. 524). The methods were carried out in accordance with the relevant guidelines and regulations of the Finish Museum of National History.

### Statistical analyses

We analysed the data with the aid of SAS System version 9.4 (SAS Institute Inc.) in three steps. First, we calculated correlations between the individual variables involved to check for possible multicollinearity (listed in Supporting Information file S1). Significant correlation was found between the number of eggs (E), hatchlings (H) and fledglings (F)—(EH: 0.86, P < 0.0001; EF: 0.44, P < 0.0001; HF: 0.54, P < 0.0001). The number of eggs, hatchlings and fledglings were redundant, according to low eigenvalues and large condition indices, so that only one of these three variables entered a statistical model. The same happened to meteorological variables temperature and total snow cover.

Second, we applied model selection based on the information-theoretic paradigm using Akaike’s Information Criterion—IT-AIC^59^ and prepared a priori multiple hypotheses based on the remaining biologically relevant variables after testing for collinearity (each model/hypothesis tested including biological explanations of applied fixed effects are listed in the Supporting Information file S1). There are warnings in the literature that Akaike Information Criterion (AIC)^60^ cannot be safely used in case of nested and mixed models^61,62^. Therefore, we used the two most important and frequent model selection criteria^62^, i.e. AIC, and Bayesian methods (BIC)^63^. Multiple information criteria are useful because each one was developed to optimize something different than the others. AIC is an example of efficient information criteria, while BIC is an example of consistent information criteria^64^. We found justification for such a procedure in a study of Posada and Buckley^65^. They argued and proved that AIC and BIC are able to simultaneously compare multiple nested or non-nested models and assess model selection uncertainty^59^.

The differences (Δ_*i*_) between the Fit statistic values (the smallest values indicating the best fitting model) were sorted according to AIC values. Akaike weight *w*_*i*_ can be interpreted as the probability that M_*i*_ is the best model (in the AIC sense, that it minimizes the Kullback–Leibler discrepancy), given the data and the set of candidate models^66^. For five models with the lowest AIC values, we therefore calculated Δ AIC, Akaike weights *w*_*i*_, and for estimating the strength of evidence in favour of one model over the other we divided their Akaike weights *w*_*min*_*/w*_*j*_ (AIC Odds)^66^. As recommended by various authors^66–68^, using the same formulas just replacing AIC by BIC values, we obtained analogically Δ BIC, BIC weights *w*_*i*_, and BIC Odds. The advantage of this is that in comparison with AIC, BIC severely penalizes models with more parameters. Thus, the BIC weights *w*_*i*_ are appreciably different than for AIC weights *w*_*i*_^67^.

Third, with the exception of breeding density estimate, clutch size, number of fledglings produced per breeding attempt, and laying date in particular analyses where these were dependent variables, fixed effects included or excluded according to alternative hypotheses within the four models (i–iv) were: year (breeding season), laying date, breeding density estimate, three categories of male and female age (1, 2 or 3 + years old), BCI of males and females, two to six months means (i.e. all adequate combinations of two, three, four, five and six months means; applied also for the two following weather variables) of daily temperature (°C), two to six months means of total snow cover (cm), two to six months means of daily precipitation (mm), and abundance of main prey (voles) in current spring or previous autumn. Since we were mostly interested in the long-term changes of the dependant variables describing our study population, the fixed effect “year (breeding season)” was included in every single model (i–iv). All relevant data used in the analyses is presented in the Supporting Information file S1.

Having the best composition of all models according to IT-AIC (models i–iv, Table 1), these we finally calculated using Generalized Linear Fixed Model (GLM, PROC MIXED in SAS, models i and iii), or General Linear Mixed Model (GLMM, PROC MIXED in SAS, models ii and iv). Using four different models (i–iv), we assessed associations between (i) breeding density estimate (i.e. number of nests/100 nest boxes), (ii) clutch size, (iii) number of fledglings produced per breeding attempt, and (iv) laying date, and fixed and random effects (see above). We performed all analyses (models ii and iv) using mixed model analysis with the individual nest, male and female as a random factor to account for the use of repeated measures on the same individuals. We estimated associations between the dependent variable and fixed effects (models i–iv) by fitting a random coefficient model using PROC MIXED as described by Tao et al.^69^.

**Table 1:** Composition of the best models.

Model	AIC	Δ AIC	AIC weights *wi*	AIC odds	BIC	Δ BIC	BIC weights *wi*	BIC odds
**Model (i)—number of nests/100 nest boxes**								
Year autumn prey abundance temperature (October–March)	76.82	0.00	0.42	1.00	78.54	0.00	0.42	1.00
Year autumn prey abundance	77.18	0.36	0.35	1.20	78.92	0.39	0.35	1.21
Year autumn prey abundance autumn prey abundance*temperature (October–March)	78.28	1.46	0.20	2.07	80.00	1.46	0.20	2.07
Year autumn prey abundance*temperature (October–March)	81.94	5.12	0.03	12.94	83.68	5.14	0.03	13.10
Year spring prey abundance temperature (October–March)	91.58	14.76	0.00	1600.18	93.32	14.78	0.00	1619.58
**Model (ii)—clutch size**								
Year laying date spring prey abundance snow cover (January–March)	− 999.80	0.00	1.00	1.00	− 991.43	0.00	1.00	1.00
Year laying date spring prey abundance	− 958.76	41.04	0.00	815,995,565.9	− 950.39	41.04	0.00	815,995,496.9
Year laying date female BCI spring prey abundance snow cover (January–March)	− 848.67	151.13	0.00	6.56679E+32	− 840.39	151.04	0.00	6.26892E+32
Year laying date female age spring prey abundance snow cover (January–March)	− 847.17	152.63	0.00	1.39121E+33	− 838.87	152.56	0.00	1.34531E+33
Year spring prey abundance snow cover (January–March)	− 823.19	176.61	0.00	2.24044E+38	− 814.75	176.68	0.00	2.31897E+38
**Model (iii)—number of fledglings per initiated breeding attempt**								
Year spring prey abundance precipitation (April–June) temperature (April–June)	108.37	0.00	0.57	1.00	110.08	0.00	0.58	1.00
Year spring prey abundance temperature (April–June)	109.69	1.32	0.30	1.94	111.43	1.34	0.30	1.96
Year spring prey abundance precipitation (April–June)	112.60	4.23	0.07	8.30	114.34	4.26	0.07	8.40
Year spring prey abundance	113.86	5.49	0.04	15.55	115.62	5.54	0.04	15.93
Year spring prey abundance precipitation (April–June)*temperature (April–June)	114.88	6.51	0.02	25.85	116.61	6.53	0.02	26.15
**Model (iv)—laying date**								
Year male age female BCI autumn prey abundance snow cover (January–March)	8429.43	0.00	0.51	1.00	8437.58	0.00	0.51	1.00
Year female BCI male BCI autumn prey abundance snow cover (January–March)	8430.64	1.20	0.28	1.83	8438.78	1.20	0.28	1.82
Year male age female age autumn prey abundance snow cover (January–March)	8431.28	1.84	0.20	2.51	8439.43	1.85	0.20	2.52
Year male age female age autumn prey abundance	8506.72	77.29	0.00	6.065E+16	8514.88	77.29	0.00	6.079E+16
Year male age female BCI autumn prey abundance	8518.33	88.90	0.00	2.012E+19	8526.48	88.90	0.00	2.012E+19

Composition (applied fixed effects) of the five best fitting models sorted according to fitting statistics (the smaller the better), AIC, Δ AIC, and BIC, Δ BIC for all four modelled dependent variables (models i–iv). The following fixed effects were log-transformed before the analyses: autumn and spring prey abundance and each weather variable.

Previous studies do not provide comprehensive base for formulating reasonable a priori hypotheses predicting what part of the weather might affect, for instance, laying date in the Tengmalm’s owls and for how long period before it^42^. Therefore, a set of different time periods was tested within particular IT-AIC analyses covering two to six months means of each weather variable (daily temperature, total snow cover, and daily precipitation).

## Results

In total, we recorded 1761 nests during 1973–2018 and analysed 38 ± 36 nests each year (mean ± SD; range 2–138 nests). There were 29 ± 30 successful nests each season (range 1–121 nests) with 105 ± 121 fledglings (range 2–563 fledglings; 2.3 ± 1.0 fledglings per initiated breeding attempt, range 0.4–4.3 fledglings).

Table 1 shows five best fitting models sorted according to the fitting statistics (starting with the smallest value) for the four dependent variables tested (models i–iv). In all cases Δ AIC, AIC weights *wi* and AIC Odds revealed comparable if not even identical results with Δ BIC, BIC weights *wi* and BIC Odds. This strengthened the credibility of the results.

### Breeding density estimate (i)

For the dependent variable breeding density estimate (number of nests/100 nest boxes), the Δ AIC and Δ BIC values nominated three best fitting models covering similar combination of fixed effects (Table 1). The probability for being the correct model was analogous for these three combinations (42% vs 35% vs 20%). The best fitting model was 1.2 and 2.07 times (odds) more likely to be the correct model. On the other hand, the probability for being the correct model for the fourth best fitting model was very low (3%) having odds about 13 times against it being the correct model. Therefore, the fourth and all the subsequent models did not need to be considered.

**Figure 2:**
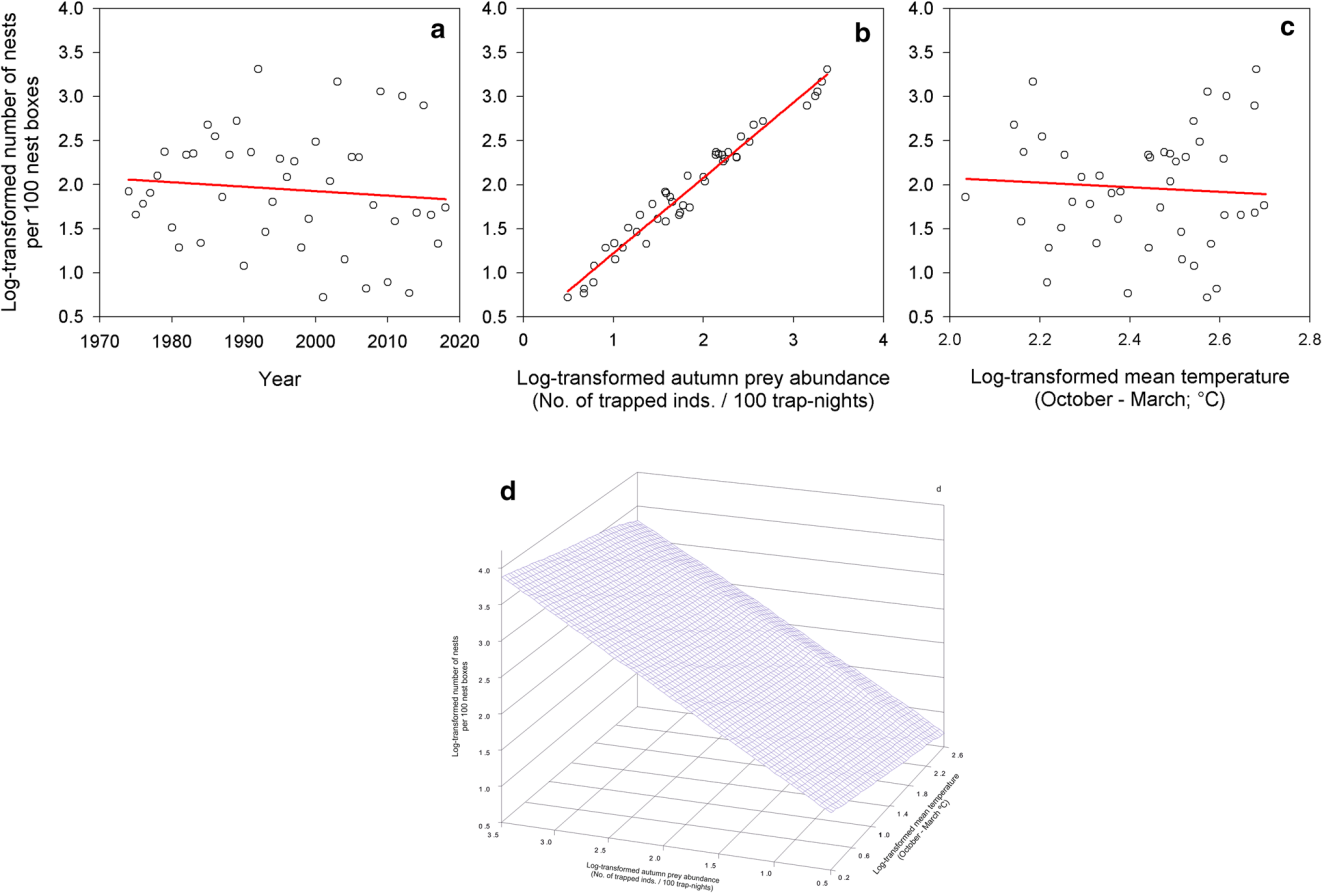
Breeding density estimate. Predicted values of breeding density estimate (log-transformed number of nests/100 nest boxes) of Tengmalm’s owls within the studied population in Kauhava region, plotted against year (**a**), log-transformed abundance index of main prey in the previous autumn (**b**), log-transformed mean temperature during the preceding October–March (**c**), and interaction between log-transformed abundance index of main prey in the previous autumn and log-transformed mean temperature during October–March (**d**).

The best model explaining breeding density estimate of the studied population included year, log-transformed main prey abundance in the previous autumn and log-transformed mean temperature during the preceding October–March (Table 1). The two competitive models included year and log-transformed main prey abundance in the previous autumn; the third one contained also interaction between log-transformed main prey abundance in the previous autumn and log-transformed mean temperature during the preceding October–March (see Table 1). The number of nests decreased over the study period (Fig. 2a, Estimated slope [ES] = − 0.01, Standard Error [SE] = 0.01, degrees of freedom [DF] = 41) but increased with increasing log-transformed abundance of main prey in the previous autumn (Fig. 2b, ES = 0.87, SE = 0.10, DF = 41). Finally, the number of nests decreased with increasing log-transformed mean temperature during the previous October–March (Fig. 2c, ES = − 0.03, SE = 0.46, DF = 41). Graphical representations of relationships in both competitive models were very similar to figures presented in Fig. 2a,b, and thus only the figure for the above-mentioned interaction is presented here (Fig. 2d).

### Clutch size (ii)

The combination of factors of the model with the lowest AIC and BIC values for the clutch size had absolute support with the probability of 100% that it is the correct model (Table 1). The model with the second lowest AIC and BIC values had odds over 10^9^ times against it being the correct model as compared to the best model in the candidate set. Therefore, the second and all the subsequent models did not need to be considered.

**Figure 3:**
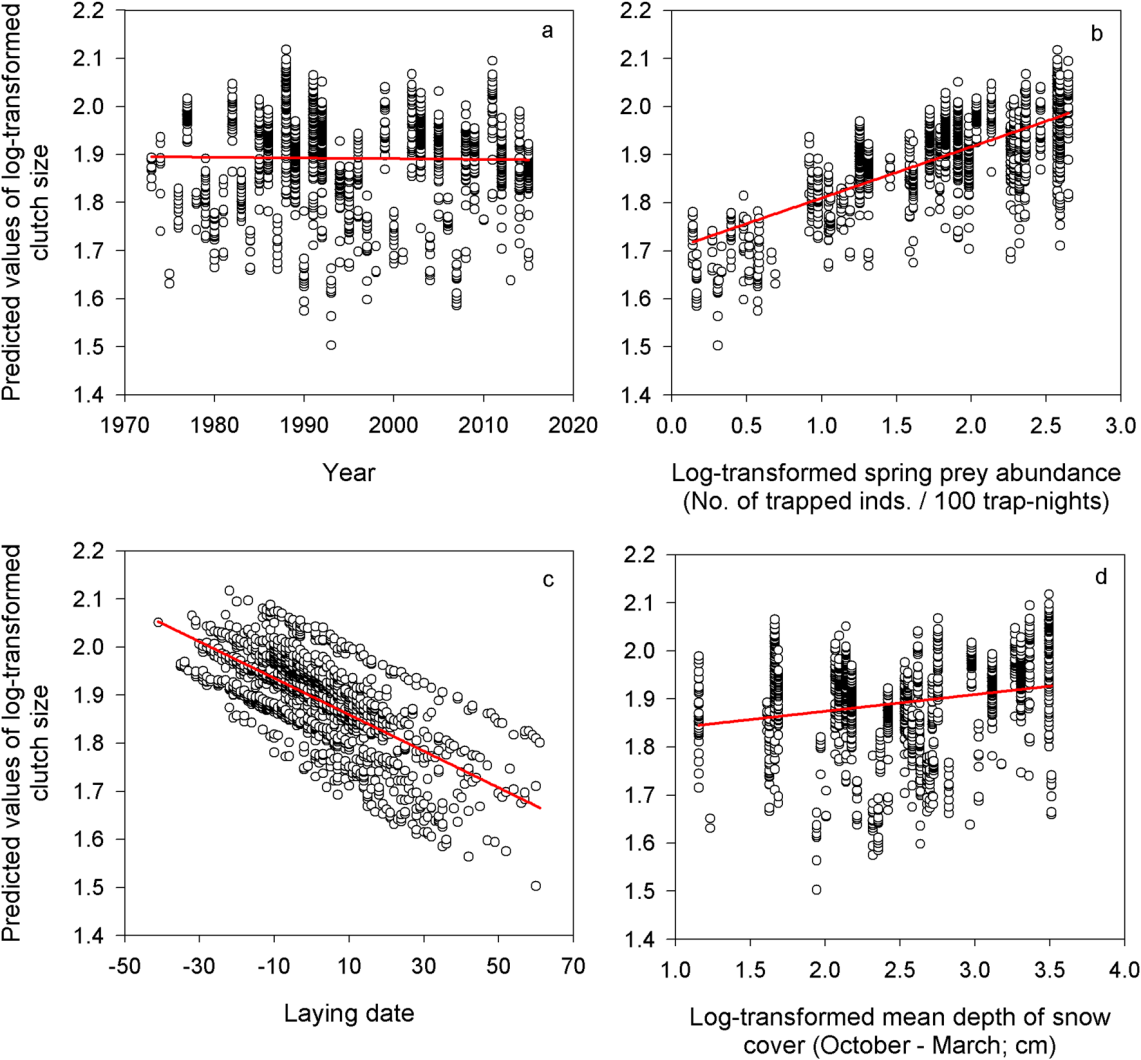
Clutch size. Predicted values of log-transformed clutch size laid by Tengmalm’s owls during breeding within the studied population in Kauhava region, plotted against year (**a**), log-transformed abundance index of main prey in the current spring (**b**), laying date (**c**; 31 March = 0, 1 April = 1, etc.), and log-transformed mean depth of snow cover during October–March (**d**).

The best model explaining final clutch size included year, laying date, log-transformed main prey abundance in the current spring and log-transformed mean depth of snow cover during the previous October–March (Table 1). Clutch size remained constant during 1973–2018 (Fig. 3a, ES = − 0.0005, SE = 0.00, DF = 1379), increased with log-transformed main prey abundance in the current spring (Fig. 3b, ES = 0.10, SE = 0.01, DF = 1405) and decreased with delayed laying date (Fig. 3c, ES = − 0.004, SE = 0.00, DF = 1415). Finally, clutch size augmented with the log-transformed mean depth of snow cover during the previous October–March (Fig. 3d, ES = 0.05, SE = 0.01, DF = 1398).

### Fledgling production (iii)

For the dependent variable the number of fledglings per initiated breeding attempt, the Δ AIC and Δ BIC values nominated two best fitting models covering similar combination of fixed effects (Table 1). The probability for being the correct model was analogous for these two combinations (57% vs 30%). The best fitting model was 1.94 times (odds) more likely to be the correct model. On the other hand, the probability for being the correct model for the third best fitting model was very low (7%) having odds about 8.3 times against it being the correct model. Therefore, the third and all the subsequent models did not need to be considered.

**Figure 4:**
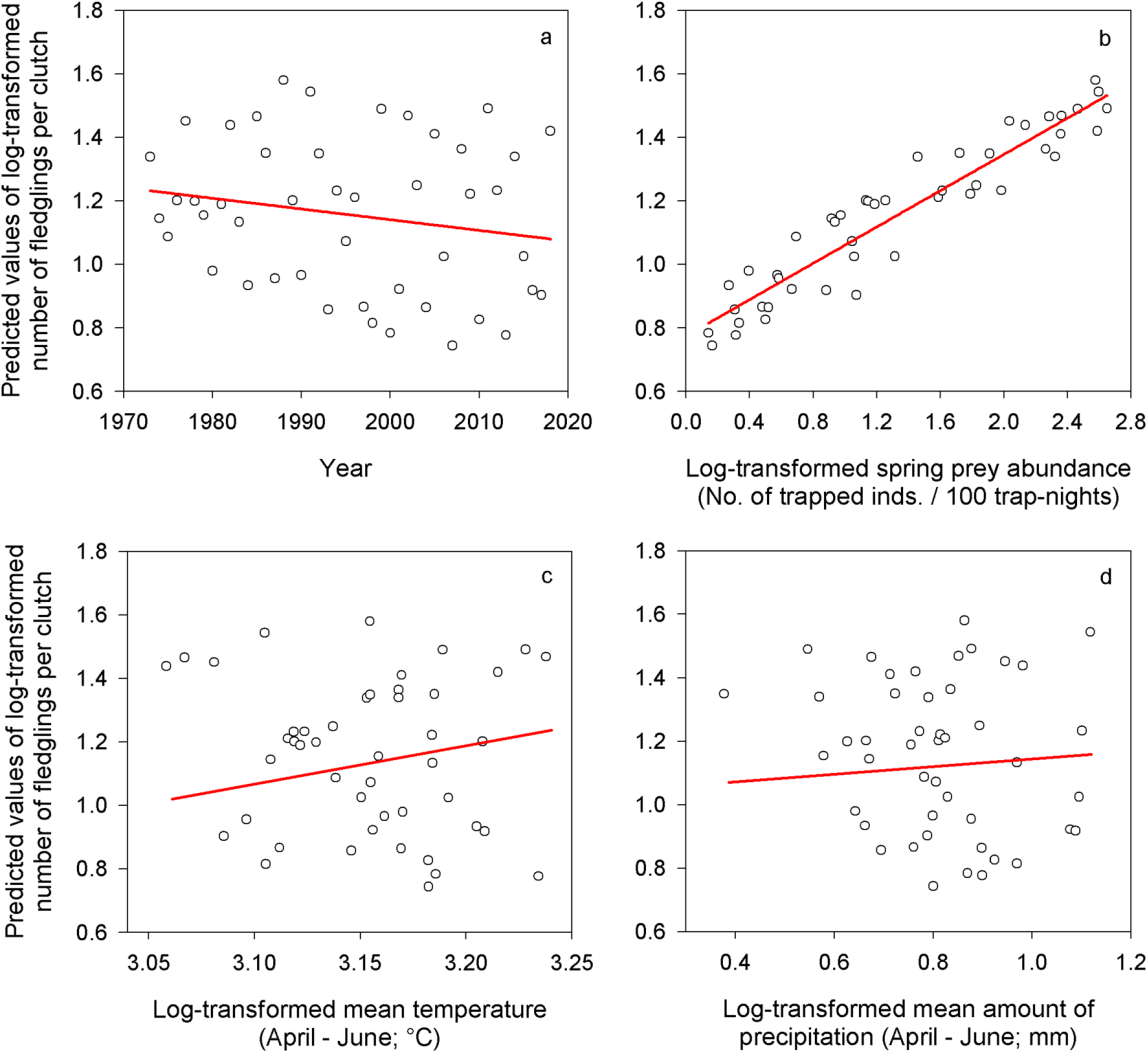
Fledgling production. Predicted values of log-transformed number of fledglings raised per breeding attempt by Tengmalm’s owls during breeding within the studied population in Kauhava region, plotted against year (**a**), log-transformed main prey abundance in the current spring (**b**), log-transformed mean temperature during April–June (**c**), and log-transformed mean amount of precipitation during April–June (**d**).

The best model explaining the number of fledglings per initiated breeding attempt included year, log-transformed main prey abundance in the current spring, and log-transformed mean temperature and amount of precipitation during April–June (Table 1). The competitive model included year, log-transformed main prey abundance in the current spring, and log-transformed mean temperature during April–June (see Table 1). The number of fledglings per clutch decreased over the study period (Fig. 4a, ES = − 0.01, SE = 0.00, DF = 41), and increased with log-transformed main prey abundance in the current spring (Fig. 4b, ES = 0.30, SE = 0.04, DF = 41), log-transformed mean temperature during April–June (Fig. 4c, ES = 0.51, SE = 0.89, DF = 41), and log-transformed mean amount of precipitation during April–June (Fig. 4d, ES = 0.01, SE = 0.24, DF = 41). Graphical representations of relationships in the competitive model were very similar to Fig. 4a–c, and are thus not repeated here.

### Laying date (iv)

For the dependent variable laying date, the Δ AIC and Δ BIC values nominated three best fitting models covering similar combination of fixed effects (Table 1). The probability for being the correct model was analogous for these three combinations (51% vs 28% vs 20%). The best fitting model was 1.83 and 2.51 times (odds) more likely to be the correct model. On the other hand, the probability for being the correct model for the fourth best fitting model was completely negligible having odds about 10^16^ times against it being the correct model. Therefore, the fourth and all the subsequent models did not need to be considered.

**Figure 5:**
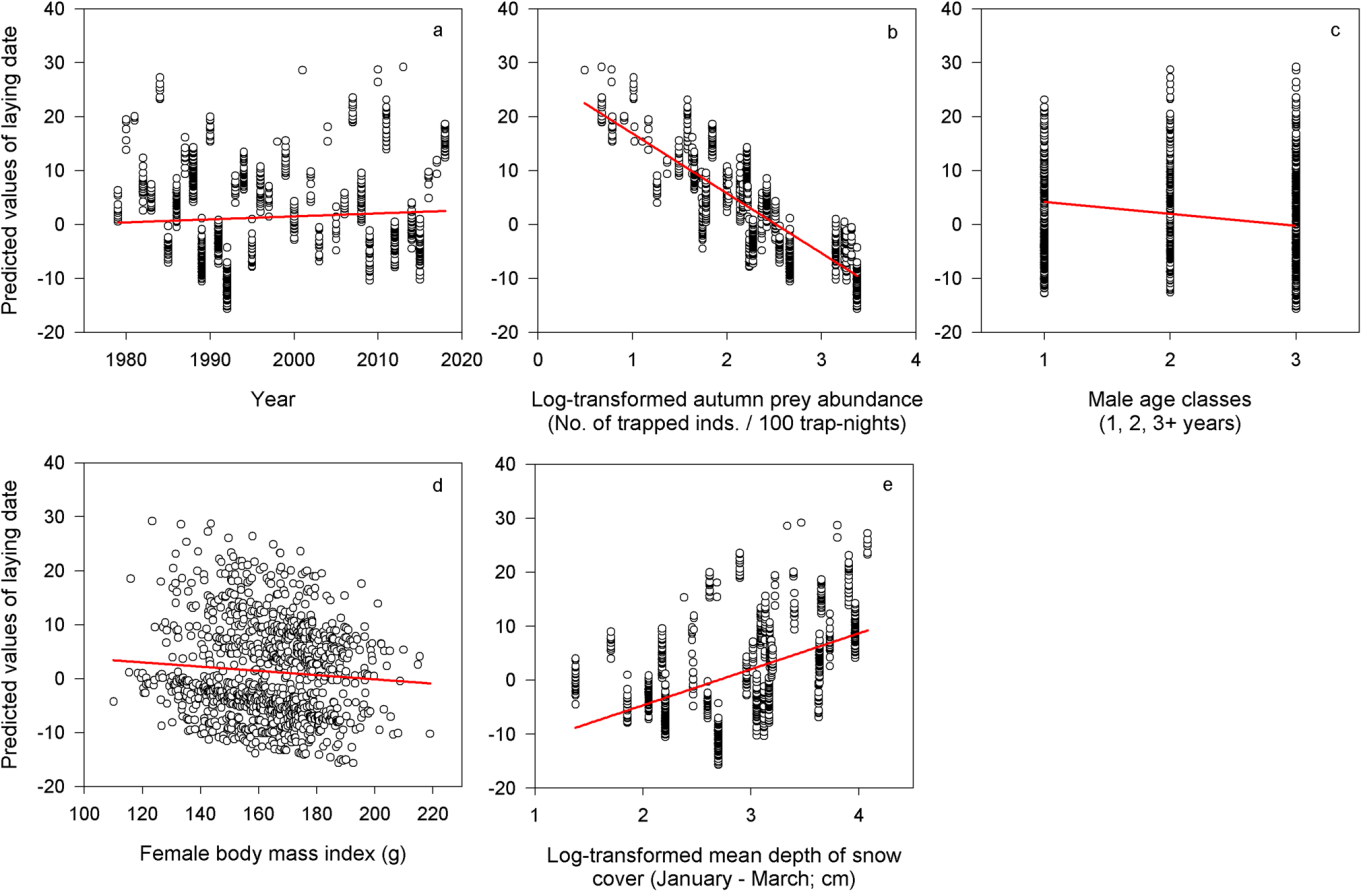
Laying date. Predicted values of laying date (31 March = 0, 1 April = 1, etc.) of Tengmalm’s owl population studied in Kauhava region, plotted against year (**a**), log-transformed abundance index of main prey in the previous autumn (**b**), age classes of male parents (**c**), body condition index of female parents (**d**), and log-transformed mean depth of snow cover during January–March (**e**).

The best model explaining laying date within the studied population included year, age of male parent, BCI of a female parent, the log-transformed abundance of main prey in previous autumn and log-transformed mean depth of snow cover during preceding January–March (Table 1). The two competitive models included BCI of males instead of male age, and female age instead of BCI of females compared to the best model (see Table 1). The laying date was slightly delayed throughout the study period (Fig. 5a, ES = 0.07, SE = 0.04, DF = 1077) but advanced with increasing log-transformed abundance of main prey in the previous autumn (Fig. 5b, ES = − 11.54, SE = 0.73, DF = 1087), age of male parents (Fig. 5c, ES = − 1.30, SE = 0.56, DF = 1087), and BCI of female parents (Fig. 5d, ES = − 0.10, SE = 0.03, DF = 1087). Finally, the laying date was delayed with increasing log-transformed mean depth of snow cover in the preceding January–March (Fig. 5e, ES = 6.81, SE = 0.70, DF = 1089). Graphical representations of relationships in both competitive models were very similar to figures presented in Fig. 5a–e (including figures for male and female age and BCI), and are thus not repeated here.

## Discussion

The main findings of this study were that fledgling production per breeding attempt showed declining long-term trend and laying date of Tengmalm’s owl population was delayed during 1970s–2010s. In addition, ambient temperature and depth of snow cover emerged as the important weather variables modifying the number of nests and/or breeding performance.

The breeding density estimate of Tengmalm’s owls declined during 1973–2018, which is consistent with earlier results in our study population and nation-wide in Finland^42,55,70^. The number of nests per 100 nest boxes inspected is highly positively correlated with the number of nests per 100 square km in Tengmalm’s owls occupying nest boxes (see Fig. 13.1. in^42^). Therefore, the density estimate used in this study corresponds well with the nest box breeding density of Tengmalm’s owls. Our results indicated that the population decline of Tengmalm’s owls is at least partly attributable to declining reproductive success (offspring production) because the number of fledglings per breeding attempt was declining during 1973–2018. The main reason for declining fledgling production was starvation of owlets during the nestling period^42^. In addition, using radio-tracking of fledglings it was found that the mortality rate of fledglings can reach up to 81% during the post-fledging dependence period only in the decline phase of 3-year population cycle of voles and this high mortality rate is mainly attributable to starvation (M. Kouba and E. Korpimäki, unpublished data). Although additional radio-tracking data from fledglings is still needed in the increase phase of the vole cycle, these results indicate that nestling and juvenile survival of Tengmalm’s owls is reduced from 1970 to 2010s. The over-winter survival and life-time reproductive success of adult male Tengmalm’s owls inhabiting home ranges containing larger proportions of mature and old-growth forest was higher than those of occupying home ranges with small proportions of these forests^48,50^.

We suggest that the reason for the decline of the Tengmalm’s owl population is due to the decreasing area of mature and old-growth forests. The males prefer to hunt in spruce and pine forests and avoid hunting in open clear-cut and agricultural areas^42,71–73^. The loss of mature and old-growth forests reduces the densities of important prey species of Tengmalm’s owls (bank voles, shrews and small birds^42^). Bank voles are the main prey of Tengmalm’s owls, particularly in winter, because they are easier to capture since they move more on the snow surface than *Microtus* voles and even climb trees^42^. Bank voles thrive in bilberry (*Vaccinium myrtillus*) rich mature and old-growth spruce-dominated forests^74,75^ because their main foods includes leaves and berries of bilberry^76^. The coverage of bilberries on the understory of coniferous forests in Finland has decreased by 50% from the 1950s to 1990s due to clear-cutting and planting pine saplings to earlier old-growth spruce-dominated forests^77^, and the decrease in coverage has continued in 2000s^78^. Because clear-cutting of old-growth forests in our study area has been going on from the 1970s to 2010s, food resources of bank voles have probably declined, which has likely had a cascading effect on predators subsisting on bank voles, including Tengmalm’s owls. In addition, willow and crested tits (*Poecile montanus*, *Lophophanes cristatus*) and other small forest birds are important alternative prey for Tengmalm’s owls, particularly in winter^42^. Population densities of willow and crested tits have declined by approx. 70–80% in Finnish forests during the study period^8,79^. Old-growth forests offer sheltered habitats against larger intra-guild predators such as Ural owls, tawny owls and goshawks^39,42^, and forest fragmentation makes movements between forest patches quite risky due to predation^80^. Interactive or additive effects of reduced food resources and increased avian predation risk most probably due to loss and fragmentation of mature and old-growth forests (both identified as crucial issues for other birds of prey^80–82^) decreases both the juvenile and adult survival of Tengmalm’s owls, leading to a long-term decrease of the population, as in the case of Northern saw-whet owls *Aegolius acadicus* in Canada^80^.

Consistent with earlier studies^42^, the number of nests increased with abundance of main food supply (voles) in the previous autumn and clutch size and number of fledglings produced per clutch augmented with vole abundance in the current spring. Similar relationships for breeding density estimates, clutch size and number of fledglings have been described many times in different bird of prey species including Tengmalm’s owls^83–87^. However, despite the negligible changes in vole cyclicity in the study area^88^, persistent decrease in number of fledglings produced at the population level throughout 1973–2018 appeared to be one of the serious threats to the persistence of the entire Tengmalm’s owl population in our study area.

Laying date of the study population advanced with increasing abundance of main foods in the previous autumn as well as with body condition of male and female parents and parental age as revealed by the three competitive models regarding laying date (Table 1). The result from vole abundance corresponds with previous ones found in different raptor and owl species including Tengmalm’s owls^24,28,83,86,89–91^. Similarly, it was shown that adults in better body condition are usually the earliest breeders^86,92–94^ and that more experienced adults (in our case + 2-year old) breed earlier in the season^42^. To nest early in the season is advantageous for several reasons. Firstly, cases of re-mating for a new breeding attempt including polygyny and successive polyandry are more frequent in early breeding individuals and are connected with higher fitness^42,95–97^. Secondly, early breeding individuals have more time to recover from breeding including time for moulting and regain condition for wintering or migration^98,99^. Thirdly, offspring of these adults could have a direct advantage due to earlier dispersion which is connected with better chance of finding suitable territory, getting more experiences before wintering and higher probability of breeding during the next breeding season^100–104^.

The start of egg-laying in Tengmalm’s owls was slightly delayed from 1970 to 2010s, which is in stark contrast to the earlier studies on passerines and other birds of prey for which earlier nesting associated with global warming due to climate change was usually recorded^18,51,105^. The probable reason is that laying date is advanced with an increasing abundance of main prey (voles) and the prime habitat of main prey (bank voles) has declined during the study period due to extensive clear-cutting of mature and old-growth forests and planting even-aged forests (see above). This interpretation is supported by the fact that weather variables did not explain the delayed laying dates in the course of the study period, because laying dates were delayed with increasing snow cover in late winter (January–March) and depth of snow cover during this period (1973–2018) tended to decline. In addition, hatching dates in Tengmalm’s owls in the nation-wide ringing data in Finland were not related to temperature in either of the three months before and during the egg-laying period (February to April^24^). This suggests that higher temperatures due to global warming were not involved in the possibly advanced start of breeding as was found, for instance, in common buzzards^23^ and goshawks^51^, and many passerines^18,105^.

The laying dates were delayed and the clutch size augmented with increasing mean depth of snow cover between January–March and October–March, respectively. These contradictory results from the effects of snow cover are not easy to explain. However, one can speculate that deep snow cover prior to and during the egg-laying period reduces the availability of voles for small avian predators including Tengmalm’s owls, because they cannot hunt voles below the deep snow cover which then delays the initiation of egg-laying. On the other hand, deep snow cover in the course of winter offers effective insulation for over-wintering voles that can even reproduce below the deep snow cover^106^, where the ambient temperature is relatively constantly close to zero ^o^C. Deep snow cover may thus have a positive effect on overwinter survival of voles^107–109^, and may result in higher vole densities in early spring, which in turn may induce larger clutch sizes of owls.

We could further speculate that with continued global warming, the population decline in Tengmalms’ owl will become steeper because temperature fluctuations around the freezing point will become more frequent as it is clear from temperature trends presented in the Supporting Information file S1. These temperatures may be especially harmful to overwinter survival of small mammals by alternate freezing and thawing events, i.e. due to the so-called “frost seesaw effect”^110–112^, and thus reducing overall availability of small mammals to predators.

We found that fledgling production of Tengmalm’s owls increased with higher temperatures and precipitation during the spring and early summer (April–June). The probable reason is that increased summer temperatures improved vole densities and maintained three-year high-amplitude cycles of vole populations in South and Central Finland^88^. Warm and humid spring and summer seasons have positive effects on vole densities via improved food supply of herbivorous voles, and thus increase offspring production of Tengmalm’s owls. Because summers are becoming warmer due to global warming at northern latitudes, higher summer temperatures may buffer negative effects of mild winters due to global warming on population cycles of voles and on the availability of small mammals to avian predators.

In conclusion, the decreasing trend of fledgling production, which was mainly due to starvation of nestlings, was an important factor contributing to the long-term decline of Tengmalm’s owl population in our study area. Milder and more humid spring and early summer temperatures due to global warming were not able to compensate for lowered offspring production of owls. The main reason (although partly speculative because we did not evaluate long-term changes in habitat composition) for low productivity is most probably loss and degradation of mature and old-growth forests due to clear-cutting based forest management which does not enable sufficient recovery of undergrowth, particularly bilberry after clear-cutting^88^. This results in loss of coverage of prime habitat for main (bank voles) and alternative food resources (small birds) inducing lack of food, and refuges against predators. This interpretation was also supported by the fact that start of egg-laying was delayed during 1970s–2010s although ambient temperatures increased and snow depth simultaneously did not increase prior to and during the egg-laying period. It is alarming that in similar adverse situation due to forest habitat loss and degradation in boreal zone are many forest-specialists belonging to different taxa^113–121^, and the situation may deteriorate as climate change progresses^2–4,122^, unless major measures to reverse this situation are taken and actually implemented as soon as possible.

## Supplementary information

Supplementary Information 1

Supplementary Information 2
