# Supplementary material for: Interactive influences of fluctuations of main food resources and climate change on long-term population decline of Tengmalm’s owls in the boreal forest
Source: Sci Rep. 2020 Nov 24;10:20429. doi: 10.1038/s41598-020-77531-y (PMC7687899; doi:10.1038/s41598-020-77531-y)
Supplement: Supplementary file 2 — Supplementary Information 2 [file 41598_2020_77531_MOESM2_ESM.docx]

**Supporting Information**

**S1 File. Supporting information.** Relevant data used in the analyses (model i – iv), long-term trends in weather variables and lists of all models/hypotheses tested within model selection based on the information-theoretic paradigm using Akaike’s Information Criterion including biological explanations of applied fixed effects.
